# MiR-502 is the first reported miRNA simultaneously targeting two components of the classical non-homologous end joining (C-NHEJ) in pancreatic cell lines

**DOI:** 10.1016/j.heliyon.2020.e03187

**Published:** 2020-01-18

**Authors:** Agnieszka Smolinska, Julia Swoboda, Wojciech Fendler, Markus M. Lerch, Matthias Sendler, Patryk Moskwa

**Affiliations:** a University Medicine Greifswald, Department of Internal Medicine A, Greifswald, Germany; b Department of Biostatistics and Translational Medicine, Medical University of Lodz, Lodz, Poland; c Department of Radiation Oncology, Dana-Farber Cancer Institute, Boston, MA, USA

**Keywords:** Cell culture, Cell death, DNA repair, Gene mutation, Chemotherapy, miRNA, DSB, NHEJ, PDAC, DNA repair

## Abstract

Pancreatic ductal adenocarcinoma (PDAC) is one of the deadliest cancers. Acquired inherited and/or somatic mutations drive its development. In order to prevent the formation of these mutations, precise and immediate repair of any DNA damage is indispensable. Non-homologous end-joining (NHEJ) is the key mechanism of DNA double-strand break repair. Here, we report that miR-502 targets two components in pancreatic cell lines, Ku70 and XLF of the C-NHEJ. Interestingly, we also observed an attenuated cell cycle response to gamma ionizing radiation (γ-IR) via diminished phosphorylation of checkpoint kinase 1 (Chk1) on serine 345 in these cell lines. Altogether, pancreatic cells showed increased susceptibility to γ-IR via direct inhibition of DNA double-strand break repair and attenuation of the cell cycle response.

## 1 Introduction

Pancreatic cancer is the 4^th^ leading cause of cancer-related deaths among all cancers. The vast majority of patients diagnosed with pancreatic cancer will die within a year. The dismal prognosis is attributed to late symptomatic manifestation and inherent chemoradioresistence. Despite extensive molecular research, formulation of new chemotherapeutic strategies, and surgical development, only very moderate progress has been achieved over the past decades and, thus, pancreatic cancer is expected by 2030 to become the second leading cause of cancer-related deaths [1].

Inherited mutations of DNA repair genes such as BRCA1, BRCA2, PALB2, FANCC, FANCG and ATM have been well established in the genesis of pancreatic cancer [2]. These genes are key players in homologous recombination (HR), the high fidelity mechanism of DSB repair. Further, the same genes participate in the Fanconi Anemia (FA) pathway to remove interstrand crosslinks. The somatic mutation of KRAS is almost ubiquitously present in pancreatic cancer and occurs already in the premalignant lesions of pancreas termed pancreatic intraepithelial neoplasias (PanIN). PanINs are believed to progress stepwise to overt pancreatic cancer due to the sequential accumulation of acquired mutations such as TP53, SMAD4 and CDKN2A [3, 4].

DNA damage response is a general term that encompasses the entire response of a single cell to DNA damage such as sensing of DNA breaks, concurrent arrest of the cell cycle propagation, repair of the breaks, and activation of apoptosis, or senesce; if the DNA breaks are irreparable [5]. DSBs constitute the most dangerous kind of DNA damage that occurs, for example, during replication, oxidative stress, and exposure to ionizing radiation (IR). NHEJ is considered as the major mechanism of DNA DSB repair in mammalian cells. Its activity dominates in the G0 and G1 phase and it decreases in the S and G2 phase of the cycle when HR, a high fidelity mechanism, is needed to ensure adequate and safe replication of DNA. NHEJ directly ligates broken DNA ends with minimal or no end processing, resulting in frequent mutations at the repair junctions. The C-NHEJ encompasses four highly conserved core components forming the Ku70/Ku80 and LIG4/Xrcc4 heterodimers as well as three accessory components Artemis, DNA-PKcs and XLF [6, 7]. The role of C-NHEJ in pancreatic cancer has been poorly studied, though it is the major DNA repair mechanism of DSBs, and its inhibition sensitizes pancreatic cancer cells to IR [8].

Radioresistance has been found to be associated with enhanced cell cycle response [9]. The key regulators of the cell cycle checkpoints are the Chk1 and Chk2 kinases, which are both downstream of two other kinases ATR and ATM, respectively, that are directly recruited to the site of DNA damage [10, 11]. Phosphorylation of Chk1 on S317 and S345 has been closely linked to DSB repair and the extent of S345 phosphorylation correlated with radioresistance in brain and lung cancer [9, 10, 12].

MiRNAs are single-stranded RNA molecules (about 20 nt long) that block protein translation and/or induce degradation of target mRNAs. A single miRNA possesses the unique ability to target up to hundreds of proteins and, thus, miRNAs are perfectly suitable to synchronize DNA damage response [13]. In our previous studies, we reported miR-182 to regulate the expression of a DSB protein BRCA1 in the breast cancer cells [14]. We also reported a set of four miRNAs to determine the sensitivity of glioblastoma cancer cells to γ-IR [12].

In the current work, we aim to identify miRNAs targeting NHEJ components and to establish their role in pancreatic cancer cell lines. We found miR-502 to target directly two components of classical NHEJ Ku70 and XLF. Further, we observed diminished phosphorylation of Chk1 on serine 345 (S345). As a consequence, pancreatic cells exhibited increased DNA damage and sensibility to γ-IR.

## 2 Methods

### 2.1 TaqMan based microarray

K562 cells were either synchronized with 2mM Thymidine and 100 ng/ml Nocodazole double block in M-phase or differentiated with 16nM PMA to megakaryocytes. The cells were released from the Nocodazole block with RPMI-1640 media containing 20% FBS and collected in G1 and S phase. Total RNA was extracted with Trizol (Invitrogen). Expression of miRNAs was analyzed using TaqMan Human MiRNA Array v 1.0 (Applied Biosystems).

### 2.2 Algorithmic tools to predict miR-502 targets

RNA22 (http://cbcsrv.watson.ibm.com/rna22_targets.html), RNAhybrid (http://bibiserv.techfak.uni-bielefeld.de/rnahybrid/) and PITA (http://genie.Weizmann.ac.il/pubs/mir07 /mir07_prediction.html).

### 2.3 Western blotting

A total lysate of 10–30μg protein was loaded on SDS-Gels and transferred to nitrocellulose membranes. The membranes were incubated with primary antibodies overnight and subsequently, probed with secondary HRP-conjugated antibodies. Primary antibodies: GAPDH (Life Science H86504M), Beta-Actin (Cell Signaling 4967), Ku70 (Santa Cruz sc-1486), Ku80 (Santa Cruz sc-9034), XLF (Santa Cruz sc-393844), DNA Ligase IV (Santa Cruz sc-28232), H2A.X (Cell Signaling 2595), γ-H2A.X-S139 (Cell Signaling 9718), Chk1 (Santa Cruz sc-7898), p-Chk1-Ser345 (Santa Cruz sc-17922), p-Chk1-S317 (Cell Signaling 2344), Chk2 (Santa Cruz sc-9064), p-Chk2-Thr68 (Santa Cruz sc-16297).

### 2.4 Virus preparation and transduction of pancreatic cell lines

HEK-293T cells were cotransfected with lentiviral packaging plasmids (pCMV-dR8.91, pMD2.G-VSVG) and transfer plasmids (pLVX-DsRed-Monomer-C1 expressing miR-scr or miR-502) by calcium phosphate precipitation method (CalPhos™ Mammalian Transfection Kit, Takara 631312). The supernatant containing the virus was collected 48 h late and concentrated using PEG Virus Precipitation Kit (BioCat K904-50/200). Cells were transduced with lentivirus particles in the presence of polybrene.

### 2.5 Dual Luciferase Assay

Hela cells were transfected with DNA plasmids (pLVX-DsRed, pMIR-REPORT, pRL-CMV) using Rotifect+ (Carl Roth CL21.2) and processed according to the Dual-Luciferase Reporter Assay protocol (Promega E1910). Pre-miR-502 was expressed from pLVX-DsRed-Monomer-C1 vector (Clontech) cloned downstream of the dsRED gene and 3′UTRs of Ku70, Ku80, LIG4 and XLF were fused downstream to the firefly luciferase gene in the pMIR-REPORT vector (Ambion). pRL-CMV vector was cotransfected to account for transfection efficiency and cell death (Promega).

### 2.6 Quantitive PCR

Total RNA was prepared with the RNeasy Mini Kit (Qiagen 74106) and transcribed using random hexamers and MMLV reverse transcriptase (Epicentre TR80125K). The quantitative expression of the mRNA was measured with QuantStudio 7 Flex Real-Time PCR (Life Technologies) using the SYBR Select Master Mix (Applied Biosystems) according to the manufacturers manual. Gene-specific primers:

Ku70: F:ACTGCAACACTTGAAGTCAAATCAAAG-R:GATTTTCAACTCAGGAGGCAGTTC.

Ku80: F:GATAACCATGTTTGTACAGCGACAG-R:TCAGTGCCATCTGTACCAAACAG.

LIG4: F:CTTGCGTTTTCCACGAATTGA-R:TCCAGGGTCATGCACTCATG.

XLF: F:CCTGTTCGTGTACCCAGAGGAG-R:TAGCTCCCTCACTTGGCACT.

U1: F:CCATGATCACGAAGGTGGTT-R:ATCCGGAGTGCAATGGATAA.

miR-502: F:CTAGTGCTGGCTCAATGCAA-R:CTCCCCCTCTCTGAATCCT.

### 2.7 Cell cycle analysis

PaTuT cells either expressing miR-scr or miR-502 were fixed in 70% ethanol. Subsequently, the cells were analyzed in PI/RNase Staining buffer (BD Pharmingen 550825) with the BD LSRII.

### 2.8 Cells synchronization assay

PaTuT cells were synchronized in G0 phase by depriving the cells of FBS for 48 h. For S phase, cells were released from the block by 20% FBS in DMEM media and collected at 12 h. The synchronization was confirmed by PI/RNase staining and FACS analysis.

### 2.9 Clonogenicity assay

Indicated cells (150 cells/well) were seeded on 6 well plate. Next day, cells were exposed to an indicated dose of γ-IR. Subsequently, cells were kept in culture until visible colonies occurred. The colonies were stained with crystal violet in 70% methanol. Plates were scanned and quantified by ImageJ.

## 3 Results

**Figure 1:**
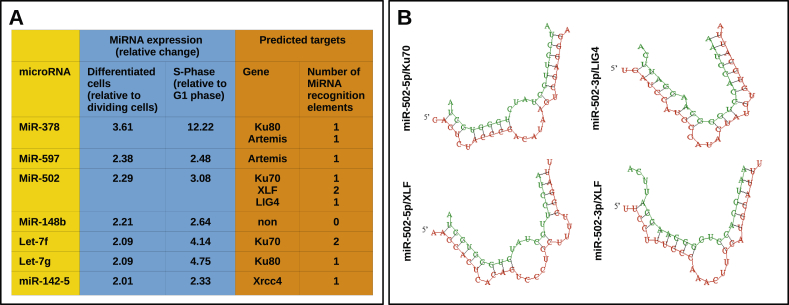
A) Summary of miRNAs identified in TaqMan microarray analysis potentially targeting components of classical NHEJ. Labeled with blue is miRNA expression in G0-and S-phase and labeled with orange are predicted targets. B) Alignment of miR-502 and its binding sites within 3′UTR of indicated proteins. The alignment was done with RNAhydrid.

Both DNA repair pathways, HR and NHEJ, are in turn actively regulated throughout the cell cycle [7]. We postulated that miRNAs constitute a possible mechanism controlling the C-NHEJ activity and, thus, their expression is expected to change throughout the cell cycle. Accordingly, we expected miRNAs inhibiting C-NHEJ to be upregulated in G0 phase due to a global decrease in the DNA repair capacity in differentiated cells (Table 1) and in S/G2 phase to favor the high fidelity repair via HR-mediated repair during replication. To find miRNAs regulating C-NHEJ proteins, we employed again K562 cells, which were successfully used in our laboratory and by others to identify miRNAs targeting DNA repair proteins [14, 15]. These cells were either differentiated to megakaryocytes or synchronized in S-phase, and miRNAs expression was analyzed with TaqMan based microarray. We found 7 miRNAs that were concurrently upregulated more than 2-fold in the G0 (differentiated cells) and in the S phase of the cell cycle (Figure 1a). Subsequently, all 7 miRNAs were subjected to computational analysis to predict their potential 3′UTR-mRNA targets. We considered each 3′UTR-mRNA as a potential target when the same miRNA binding site was predicted by all 3 applied algorithms. Indeed, we found that six out of the seven miRNAs potentially target 3′UTR-mRNA of C-NHEJ pathway but only miR-502 appeared to regulate three targets such as LIG4, Ku70, and XLF. Alignment of the miR-502 with the binding site within the 3′UTRs of mentioned targets is shown in Figure 1b.

**Table 1:** Summary of DNA repair capacity of differentiated cells.

Cell type	Repair of DSB in differentiated cells	References
Rat neurons	Lower than astrocytes	Wang, T. S. & Wheeler, K. T. Repair of X-ray-induced DNA damage in rat cerebellar neurons and brain tumor cells. Radiat. Res. 73, 464–475 (1978). Gobbel, G. T. et al. Response of Postmitotic Neurons to X-Irradiation: Implications for the Role of DNA Damage in Neuronal Apoptosis. J. Neurosci. 18, 147–155 (1998).
Mouse neuroblastoma	Decreased	Byfield, J. E., Lee, Y. C., Klisak, I. & Finklestein, J. Z. Effect of differentiation on the repair of DNA single strand breaks in neuroblastoma cells. Biochem. Biophys. Res. Commun. 63, 730–735 (1975).
Mouse 3T3-adipocyte	Decreased	Tofilon, P. J. & Meyn, R. E. Reduction in DNA repair capacity following differentiation of murine proadipocytes. Exp. Cell Res. 174, 502–510 (1988). Bill, C. A., Grochan, B. M., Vrdoljak, E., Mendoza, E. A. & Tofilon, P. J. Decreased repair of radiation-induced DNA double-strand breaks with cellular differentiation. Radiat. Res. 132, 254–258 (1992).
Mouse bone marrow	Decreased	Murray, D. & Meyn, R. E. Differential repair of gamma-ray-induced DNA strand breaks by various cellular subpopulations of mouse jejunal epithelium and bone marrow in vivo. Radiat. Res. 109, 153–164 (1987).
Human K562-erythroleukemia	Slower rate of repair	Latella, L., Lukas, J., Simone, C., Puri, P. L. & Bartek, J. Differentiation-induced radioresistance in muscle cells. Mol. Cell. Biol. 24, 6350–6361 (2004).
Chicken erythrocytes	No repair	Tabocchini, M. A. et al. Formation and repair of DNA double-strand breaks in gamma-irradiated K562 cells undergoing erythroid differentiation. Mutat. Res. 461, 71–82 (2000).
Mouse jejunum epithelium	Decreased in villi vs crypts	Karran, P. & Ormerod, M. G. Is the ability to repair damage to DNA related to the proliferative capacity of a cell? The rejoining of X-ray-produced strand breaks. Biochim. Biophys. Acta 299, 54–64 (1973).
Mouse colon epithelium	Decreased in villi vs crypts	Kulkarni, M. S. & Yielding, K. L. DNA damage and repair in epithelial (mucous) cells and crypt cells from isolated colon. Chem. Biol. Interact. 52, 311–318 (1985).

**Figure 2:**
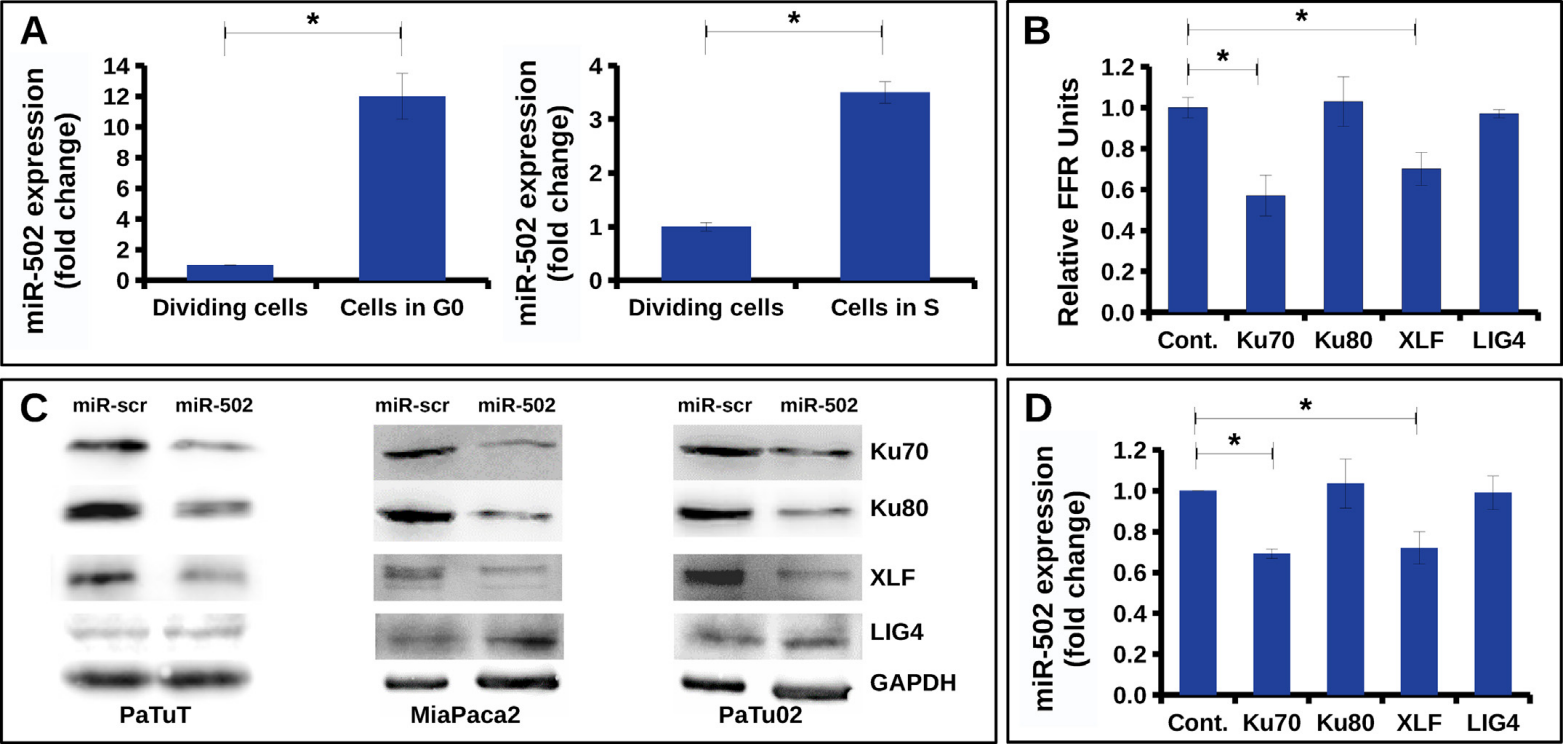
Biological validation of predicted targets. A) MiR-502 upregulation in G0 and S phase in PaTuT cells synchronized by FBS starvation (n = 3; mean ± SD; paired t-test; p < 0.05 marked with *). B) Dual luciferase assay in Hela cells (n = 3; mean ± SD, one-way ANOVA, post-hoc Dunnett's test vs controls, p < 0.05 marked with *). C) Western blot analysis of PaTuT, MiaPaca2 and PaTu02 cells overexpressing exogenous miR-502. Images of original Western blot can be found in supplementary material figure 1. D) mRNA expression of indicated proteins quantified by SyGr based qPCR (n = 3; mean ± SD; one-way ANOVA p ≤ 0.05; post hoc Dunnett's test p < 0.05 marked with *).

**Figure 3:**
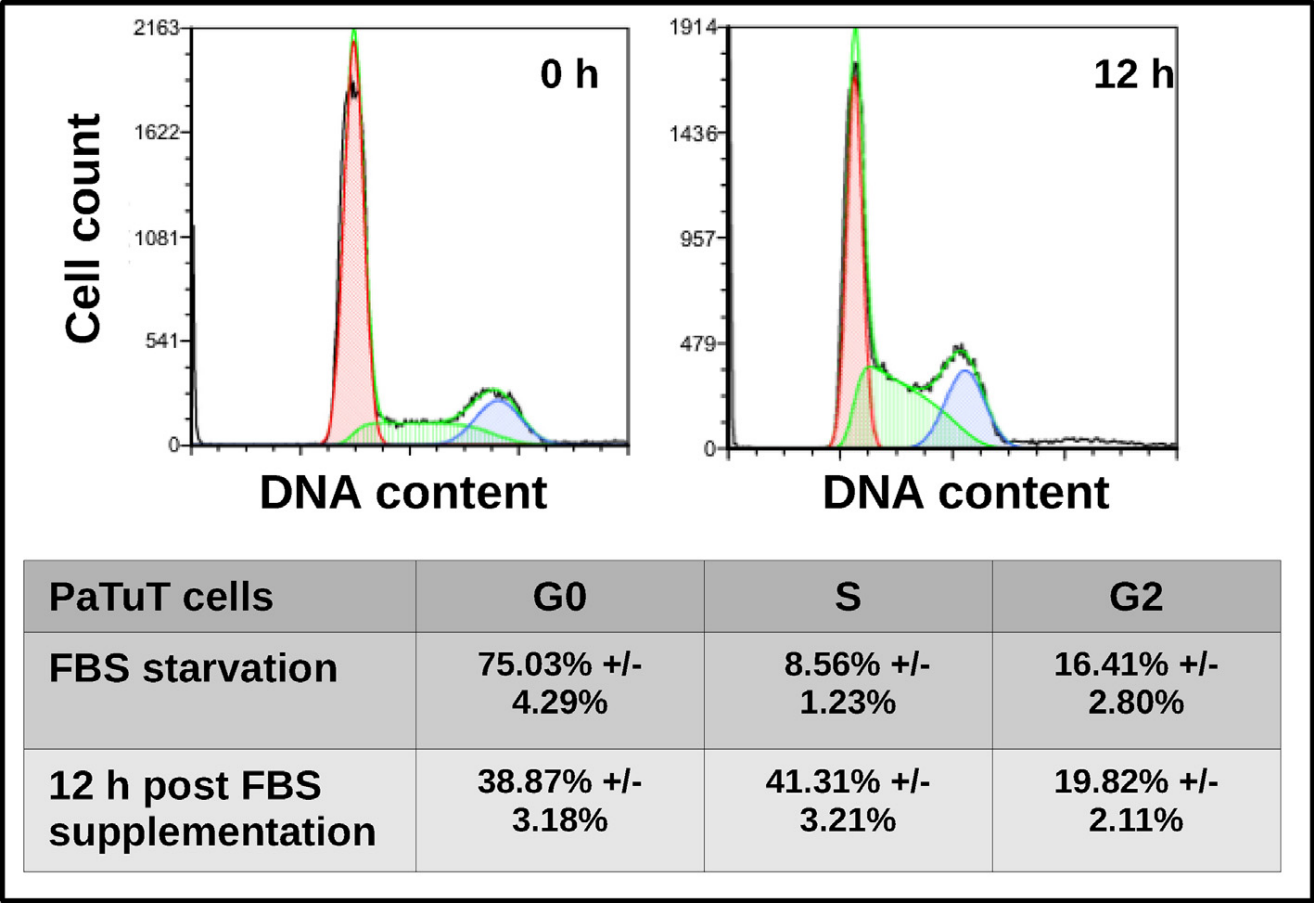
Synchronization of PaTuT cells. Cells were seeded on 6 wells plate and next deprived of FBS for 48 h. The cells were released from G0 phase of the cell cycle by 20 % FBS and collected and 0, 1, 3, 6, 12, and 24 h. The highest percentage of PaTuT cells in S phase was determined by FACS analysis at 12 h post FBS supplementation. The plots show the distribution of cells in a different phase of the cell cycle at 0 h (48 h after the beginning of FBS starvation) and 12 h (after FBS supplementation).

**Figure 4:**
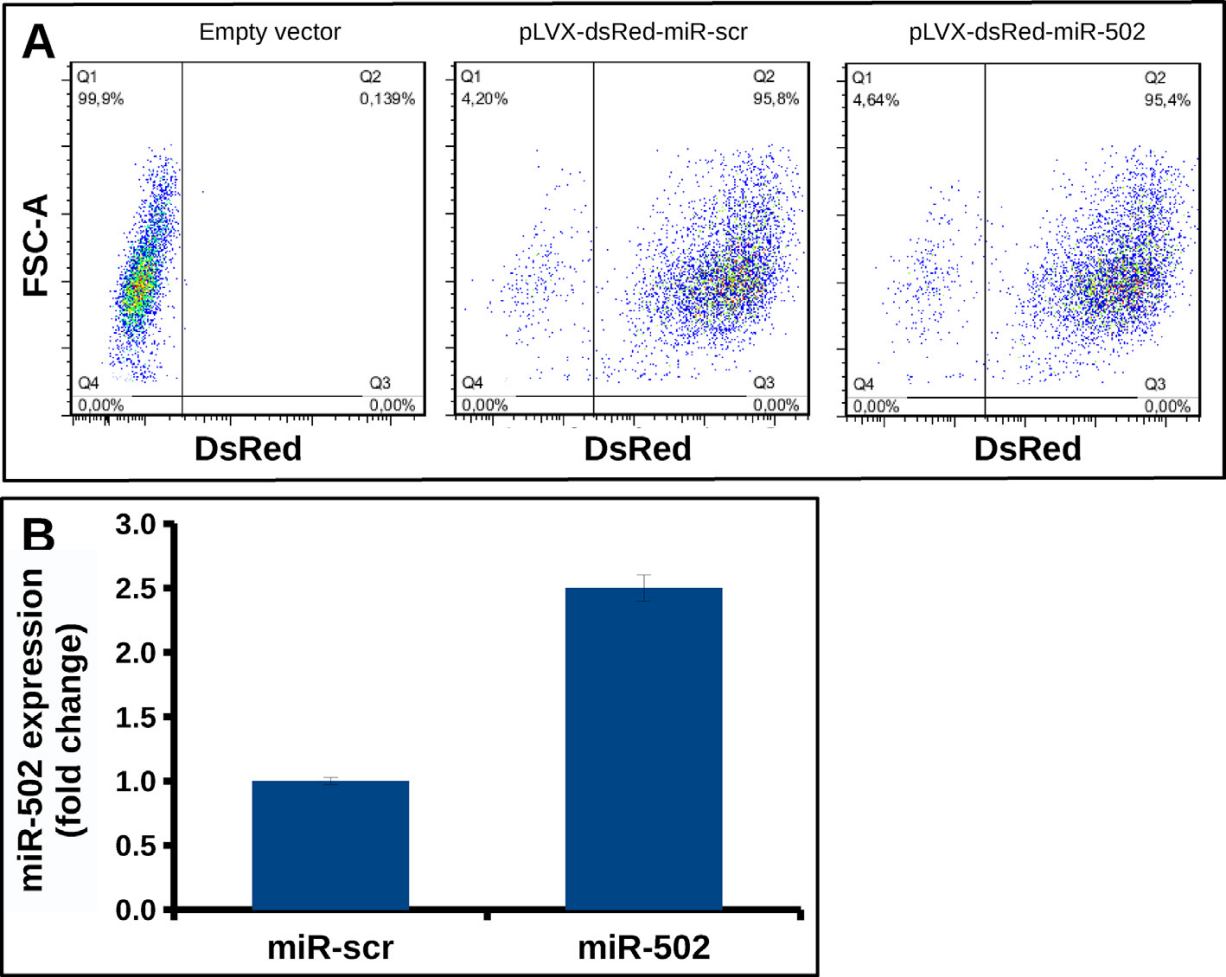
Transduction of PaTuT cells with lentivirus*.* A) The PaTuT cells were transduced with Lentivirus expression pLVX-DsRed-Monomer-C1 containing either miR-scr or miR-502 and the transduction efficiency was assessed by FACS analysis. B) Expression of miR-502 compared to miR-scr control in the PaTuT cells transduced with lentivirus.

As reported by Li *et al.*, siRNA mediated inhibition of C-NHEJ results in increased sensitivity of pancreatic cells to γ-IR [8]. Thus, we raised the question whether naturally occurring miRNAs may be potential regulators of C-NHEJ in pancreatic cells and determine their sensitivity to γ-IR. To confirm the relevance of miR-502 in pancreatic cells, we first investigated its cell cycle dependent expression in PaTuT cells. Analogously to K562 cells, we found that miR-502 was upregulated in G0 and S phase of the cell cycle (Figure 2a). The corresponding FACS analysis of the cell cycle is shown in Figure 3. Applying the dual-luciferase assay in HeLa cells, we confirmed direct interaction of miR-502 with Ku70, and XLF, but not with LIG4, and Ku80. The firefly luciferase (FFL) activity was decreased by more than 25 % compared to scrambled miRNA control (Figure 2b). Next, we applied the lentiviral system to transduce pancreatic cell lines with exogenous miR-502. In this system, the fluorescent protein dsRED was coexpressed and, as shown by FACS analysis, we achieved a transduction efficiency of 95 % in all cell lines. The expression of miR-502 increased by 2–3 fold compared to cells transduced with miR-scr control (Figure 4a and b). In line with the dual-luciferase assay results, overexpression of miR-502 in PaTuT, MiaPaca2, and PaTu02 cells decreased the expression of the endogenous level of Ku70 and XLF but not of LIG4 (Figure 2c). As expected, there was a concurrent decrease of Ku80 expression as both proteins, Ku70 and Ku80 form a heterodimer, which stability depends on mutual interaction of both proteins [16, 17]. We further validated the results in PaTuT cells applying qPCR to quantify mRNA expression levels. Again, overexpression of miR-502 decreased the mRNA expression of Ku70 and XLF but did not affect the mRNA level of LIG4 and Ku80 (Figure 2d).

**Figure 5:**
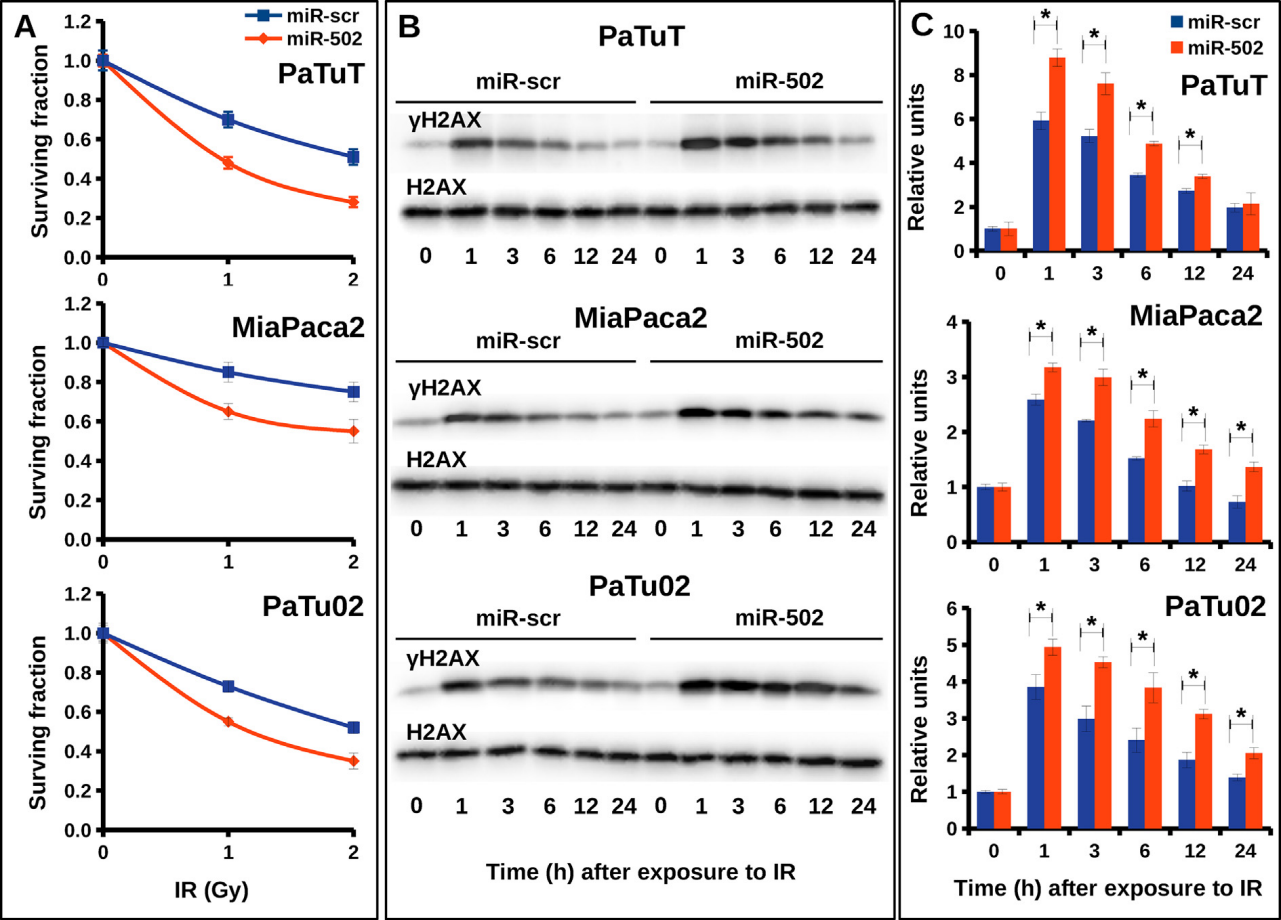
Impact of miR-502 on the survival and DNA damage in pancreatic cells exposed to γ-IR. A) Survival curve of PaTuT, MiaPaca2 and PaTu02 cells overexpressing either miR-scr or miR-502 exposed to indicated dose of γ-IR. Significant differences were confirmed between the negative control (miR-scr) and miR-502 at all doses of radiation and in the AUC (Area Under the Curve) comparison (n = 3; mean ± SD; t-test, p = 0.01). B) Representative Western blot showing expression of γ-H2AX and H2AX in PaTuT, MiaPaca2 and PaTu02 cells exposed to 10 Gy of γ-IR. Images of original Western blot can be found in supplementary material figure 2. C) Quantification of Western blots normalized to H2AX (n = 3, mean ± SD, t-test, differences with p < 0.05 marked with *).

Deficiency of C-NHEJ components results in an increased sensitivity to IR [6]. In line, shRNA mediated knockdown of Ku70 or Ku80 in pancreatic cell lines led to the reduced survival of these cells exposed to γ-IR [8]. In order to support the role of miR-502 in the regulation of C-NHEJ, we overexpressed miR-502 in PaTuT, MiaPaca2, and PaTu02 cells and exposed these cells to γ-IR. As determined by clonogenicity assay, exogenous expression of miR-502 sensitized all three cell lines to γ-IR (Figure 5a). To further support the role of miR-502 in DNA repair, we analyzed the expression of γ-H2AX post-exposure to γ-IR. We treated PaTuT, MiaPaca2 and PaTu02 cells overexpressing miR-502 with γ-IR and determined the remaining DNA damage overtime by Western blotting for γ-H2AX (Figure 5b). Quantification of three independent Western blots revealed a significant increase of the remaining DNA damage in cells overexpressing miR-502 at 1, 3, 6, 12 and 24 h post-γ-IR exposure, however, there was no significant difference at 24 h in PaTuT cells (Figure 5c).

**Figure 6:**
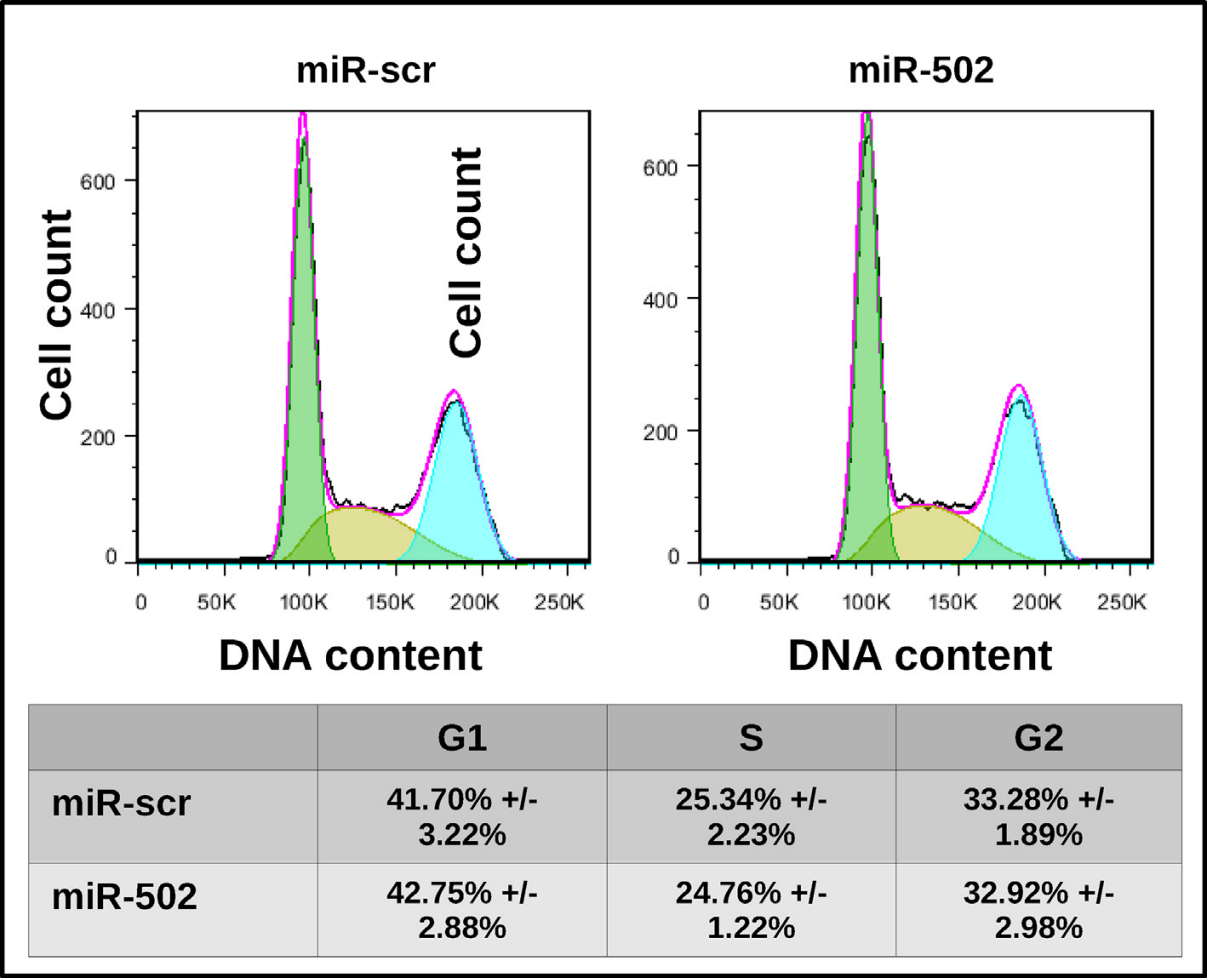
Impact of miR-502 on the cell cycle in PaTuT cells. Cells were transduced with control (miR-scr) or miR-502 and stained PI/RNase for FACS analysis.

**Figure 7:**
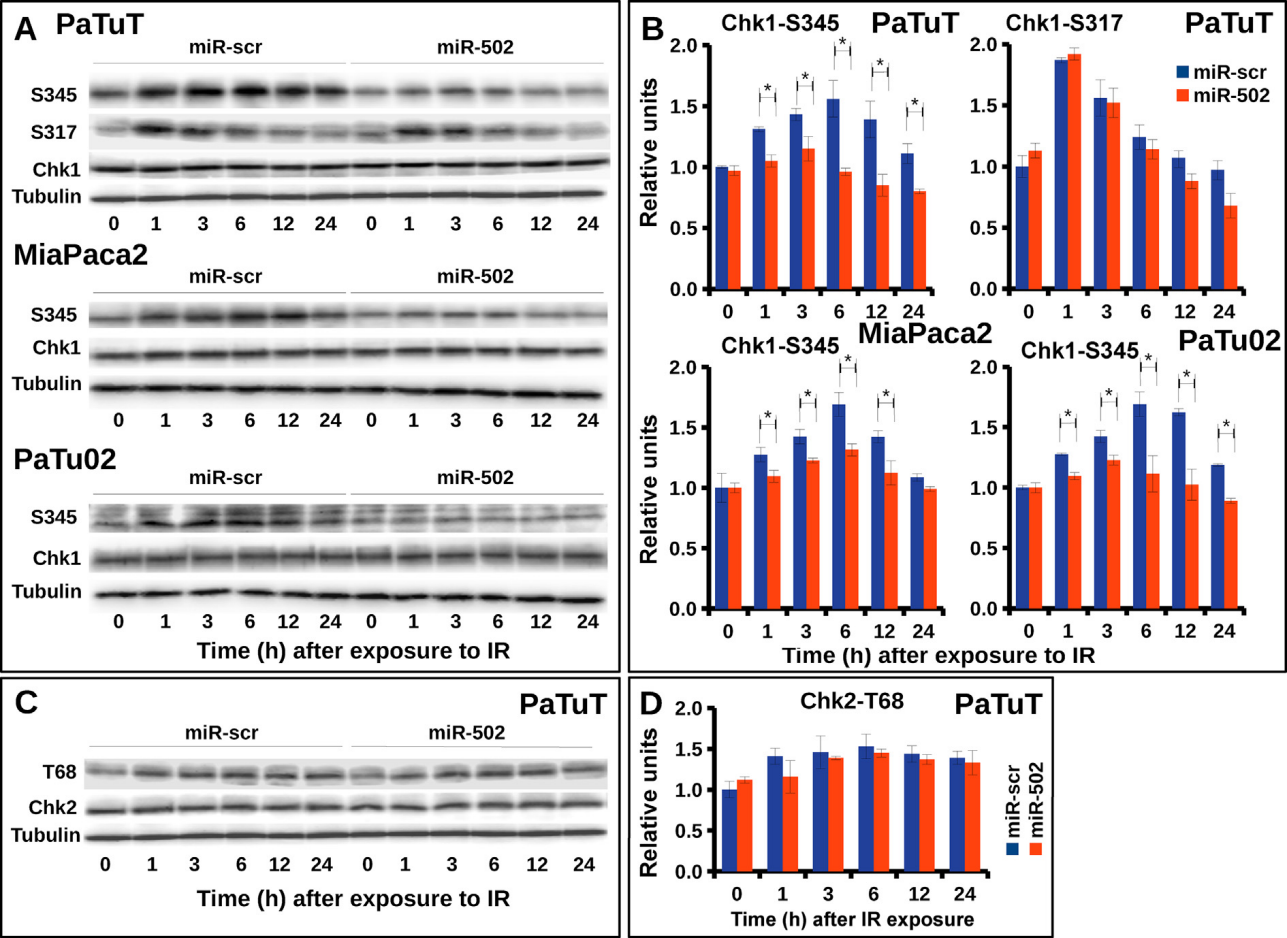
Impact of miR-502 on phosphorylation status of Chk1 and Chk2 kinase. A) Western blot analysis of Chk1-S345 in PaTuT, MiaPaca2 and PaTu02 and Chk1-S317 in PaTuT cells upon exposure to 10 Gy of γ-IR and B) qualification of Chk1-S317 and Chk1-S345 normalized to tubulin. C) Western blot analysis of Chk2-T68 and D) quantification normalized to tubulin. Statistical analysis for B and D (n = 3, mean ± SD, t-test, differences with p < 0.05 marked with *). Images of original Western blot can be found in supplementary material figure 3.

**Figure 8:**
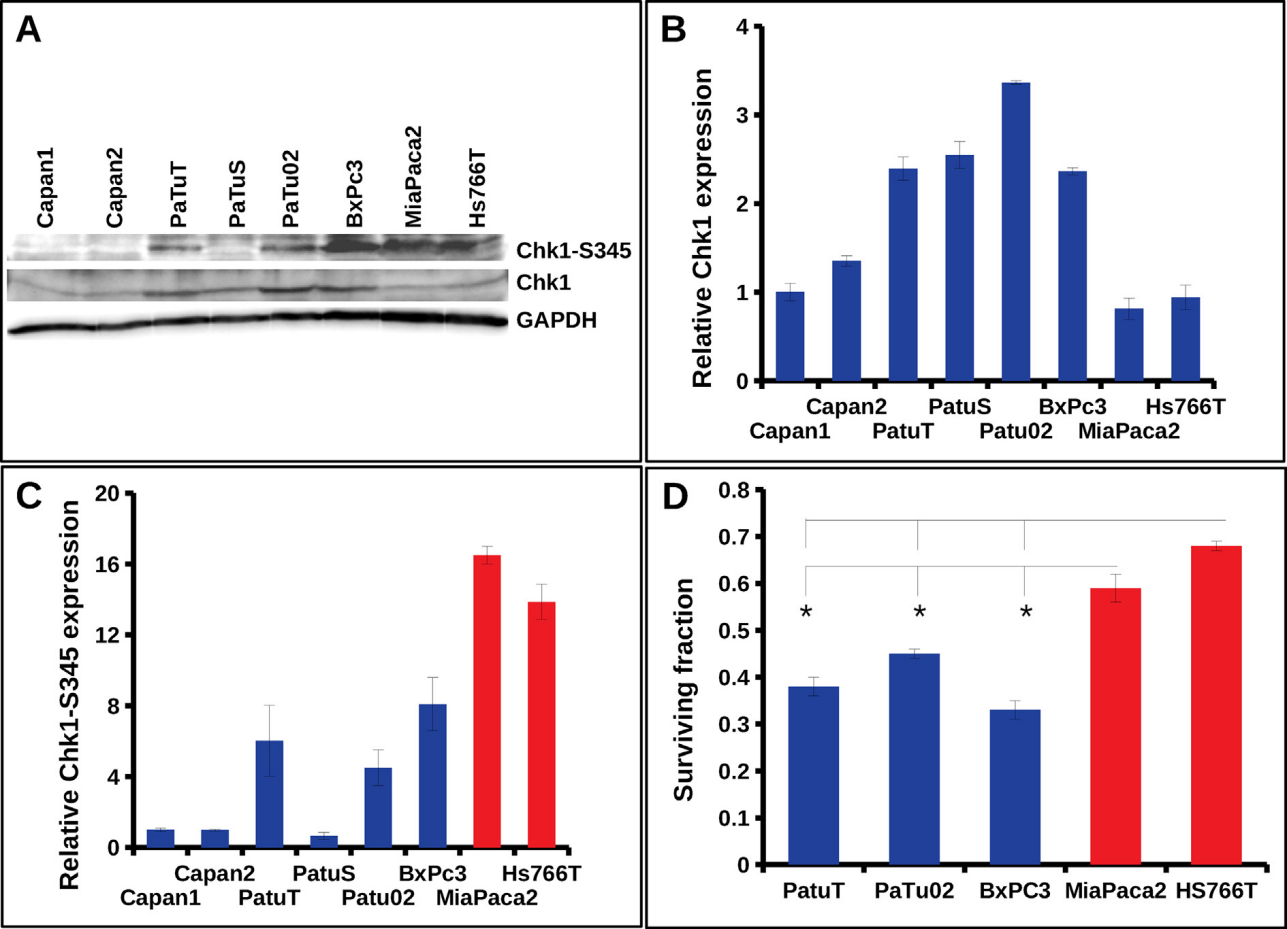
Analysis of Chk1 and Chk1-S345 expression in pancreatic cancer cell lines and correlation of Chk1-S345 phosphorylation level with γ-IR sensitivity. A) Western blots analysis of Chk1 and Chk1-S345 baseline expression in the indicated pancreatic cell lines. Quantification of Western blots showing B) the expression of Chk1 kinase relative to GAPDH and C) of Chk1-S345 relative to Chk1. Images of original Western blot can be found in supplementary material figure 4. D) Clonogenicity assay measuring sensitivity of the indicated pancreatic cell lines to 2 Gy of γ-IR (n = 3; mean ± SD; one-way ANOVA p < 0.05; differences in post-hoc Dunnett's test against controls with p < 0.05 were marked with *).

DNA damage repair and checkpoint activation occur simultaneously and both pathways are tightly synchronized with each other. Only cells, which have successfully repaired the damaged DNA, can progress to the next phase of the cell cycle [5]. MiR-502 was shown to shift cells toward G1 phase [18]. Applying flow cytometry, we analyzed the impact of miR502 on the cell cycle in PaTuT cells. To our surprise, we did not see any significant difference between the cells overexpressing miR-502 cells and the miR-scr control (Figure 6). This discrepancy may arise because the interaction of a single miRNA with its mRNA targets and the resulting biological activity is cell type and cellular context dependent [19, 20]. Nevertheless, we pursued the role of miR-502 in the cell cycle regulation because the heterodimeric Ku70/80 complex actively regulates the checkpoint activation [21]. The Chk1 and Chk2 kinases are key players in checkpoint signaling and, to exert its role, both kinases undergo during its activation extensive phosphorylation on multiple sites [6, 10, 11]. In our previous work, we demonstrated that a set of four miRNAs enhanced the phosphorylation of the Chk1 kinase on serine 345 (S345) that correlated with increased radioresistance in glioblastoma cell lines [12]. Analogously, we exposed pancreatic cells overexpressing miR-502 to γ-IR and did Western blot analysis of the key phosphorylation sites of Chk1 and Chck2 kinase. We found that phosphorylation of Chk1-S345 in PaTuT cells was clearly diminished but, in contrast, there was no difference in phosphorylation of Chk1-S317 (Figure 7a and b). We further confirmed the decreased phosphorylation of Chk1-S345 in MiaPaca02 and PaTu02 overexpressing miR-502. In all three cell lines, we observed the most pronounced effect on Chk1-S345 phosphorylation at 6 and 12 h post γ-IR exposure, whereas at 24 h we did not obtain consistent results for all cell lines. We also analyzed the phosphorylation of Chk2 kinase on the key site threonine 68 (Chk2-T68) in PaTuT cells. We did not find a significant difference in phosphorylation of Chk2-T68 (Figure 7c and d). To support the role of Chk1 in radiosensitivity, we analyzed the expression of Chk1 and the phosphorylation level of Chk1-S345 in different pancreatic cell lines. The expression of Chk1, as well as the phosphorylation level of S345, varied broadly between cell lines (Figure 8a and b). To account for the strong variability in Chk1 expression, we evaluated the ratio of Chk1 and its phosphorylated form Chk1-S345 (Figure 8c). This way, we found three cell lines Capan1, Capan2, and PaTuS to carry a very low level of the phosphorylated Chk1-S345. In contrast, MiaPaca2 and Hs766T showed a high level of Chk1-S345 phosphorylation (Figure 8c, red columns). We graded the remaining three cell lines BxPC3, PaTuT, and PaTu02 to have an intermediate level of Chk1-S345 phosphorylation. We applied the clonogenicity assay to determine the resistance of these cell lines to γ-IR. Unfortunately, due to difficult culturing conditions, we could not establish this assay for Capan1 and Capan2 cells. The PaTuS cells did not produce reliable results due to a very low (<5 %) plating efficiency (data not shown). In contrast, BxPC3, PaTuT, PaTu02, MiaPaca2 and Hs766T showed a plating efficiency of more than 60 % and provided reproducible results. We found that cells with intermediate level of Chk1-S345 phosphorylation were more susceptible to γ-IR (Figure 8d, blue columns) than their counterparts with a high level of Chk1-S345 phosphorylation (Figure 8d, red columns).

## 4 Discussion

There are different chemotherapy regimens available for pancreatic cancer, either alone or in combination with IR [22]. FOLFIRINOX (folic acid, 5-fluorouracil, irinotecan and oxaliplatin) is the most effective palliative chemotherapy for the advanced pancreatic cancer [23]. Although this regimen combines three DNA damaging drugs with three distinct mechanisms of DNA damage, the vast majority of patients become rapidly resistant to the treatment. One of the essential mechanisms of how cancer cells develop resistance has been attributed to checkpoint response. As shown by Bao et al., glioblastoma tumors, which are particularly IR resistant, exhibit an enhanced cellular checkpoint response to IR [9]. In line, we demonstrated that enhanced checkpoint response is induced by a set of four miRNAs (miR-1, miR-125a, miR-150 and miR-425) in glioblastoma cell lines and is strongly correlated with radioresistance [12].

The role of miR-502 in the genesis and progression of cancer has been fairly established [24]. One of the best-investigated targets of miR-502 is the lysine methyltransferase SET8, which monomethylates an array of proteins such as H4K20, PCNA and p53. Methylation of these proteins impacts cellular processes including cycle progression and DNA damage response [24]. Consequently, inadequate expression of SET8 due to single nucleotide polymorphism (SNP) in the miR-502 binding site has been linked to the susceptibility and outcome of different malignancies, such as breast cancer and other [25, 26, 27].

Here, we show miR-502 as a potential miRNA targeting two components of the C-NHEJ. Computational analysis revealed three possible targets such as Ku70, LIG4 and XLF. Indeed, applying biological methods, we confirmed Ku70 and XLF as direct targets of miR-502. Ku70/80 complex serves as a sensor of DNA DSBs, initiates the assembly of C-NHEJ complex, and activates DNA damage response. In contrast, XLF acts downstream of Ku70/80 complex and forms a heterotrimeric complex with Xrcc4/LIG4 that stimulates the activity of LIG4 as well as contributes to proper alignment of broken DNA ends [28]. Thus, miR-502 interferes simultaneously with the assembly of C-NHEJ complex and ligation of DNA broken ends. How Ku70/80 complex activates the DNA damage response is not exactly understood. However, binding of Ku70/80 complex to DNA DSBs induces its conformational change and enables the assembly with DNA-PKcs. In this heterotrimeric complex designated DNA-PK, the catalytic DNA-PKcs unit becomes activated and exerts extensive phosphorylation of C-NHEJ components and components of DNA damage response [29]. Tomimatsu *et al.* showed a link between Ku70/80 complex, ATM-dependent activation of ATR and phosphorylation of p53 but they did address the phosphorylation of Chk1 [30]. In another study, Zhao *et al.* demonstrated that activated ATR phosphorylates Chk1 on S317 and S345 residues [31]. Thus, we would expect that miR-502 mediated downregulation of Ku70/80 expression results in decreased Chk1 phosphorylation and attenuated cell cycle response. Indeed, we found that overexpression of miR-502 decreased phosphorylation of Chk1 kinase on residue S345, a key site in response to γ-IR, but did not affect phosphorylation of S317. In addition, we found that the extent of S345 phosphorylation correlates with radioresistance in pancreatic cell lines. The phosphorylation of Chk1 on S345 site may be a specific response to γ-IR as only this residue has been a link to radioresistance in multiple studies and its selective phosphorylation remains an interesting question for further research [9, 10, 12].

MiR-502 is, to the best of our knowledge, the first reported miRNA to target simultaneously two distinct components of the classical NHEJ. It does not only inhibits directly DNA DSB repair, but it attenuates the cell cycle response and possibly entire DNA damage response. Traditional therapeutic anticancer components are designed to target a single protein and though, some of them initially achieve a great response, they do not provide cure as the cancer cells become resistant [32]. Thus, these and other non-specific anticancer components of a distinct mechanism are combined together to improve the response and delay remission of cancer. The pleiotropic nature of miRNAs makes them excellent therapeutic candidates as a single miRNA targets multiple proteins and hits one and/or multiple pathways of the diseased cells concurrently. In the case of miR-502, we showed that two components of NHEJ are direct targets. Consequently, the DNA response in miR-502 overexpressing cells seems to be attenuated making the cells susceptible to DNA damage. We speculate, in this case cells are not only more susceptibility to DNA damage but also, because of multiple miRNA targets, acquisition of resistance may be delated or even avoided. Thus, we believe that miR-502, as well as other miRNAs, are excellent molecules for therapeutic purposes, alone or in combination with DNA damaging agents, to fight cancer cells.

## Declarations

### Author contribution statement

Agnieszka Smolinska: Conceived and designed the experiments; Performed the experiments; Analyzed and interpreted the data; Wrote the paper.

Julia Swoboda: Conceived and designed the experiments; Performed the experiments; Analyzed and interpreted the data.

Wojciech Fendler: Analyzed and interpreted the data.

Markus M. Lerch, Matthias Sendler: Conceived and designed the experiments.

Patryk Moskwa: Conceived and designed the experiments; Analyzed and interpreted the data; Wrote the paper.

### Funding statement

This work was supported by the DFG (Deutsche Forschungsgemeinschaft) grant MO 2924/1-1.

### Competing interest statement

The authors declare no conflict of interest.

### Additional information

No additional information is available for this paper.

## Appendix A Supplementary data

Supplementary material-Heliyon Supplementary material-Heliyon
