# Supplementary material for: MiR-502 is the first reported miRNA simultaneously targeting two components of the classical non-homologous end joining (C-NHEJ) in pancreatic cell lines
Source: Heliyon. 2020 Jan 18;6(1):e03187. doi: 10.1016/j.heliyon.2020.e03187 (PMC7002776; doi:10.1016/j.heliyon.2020.e03187)

Supplementary figure 1

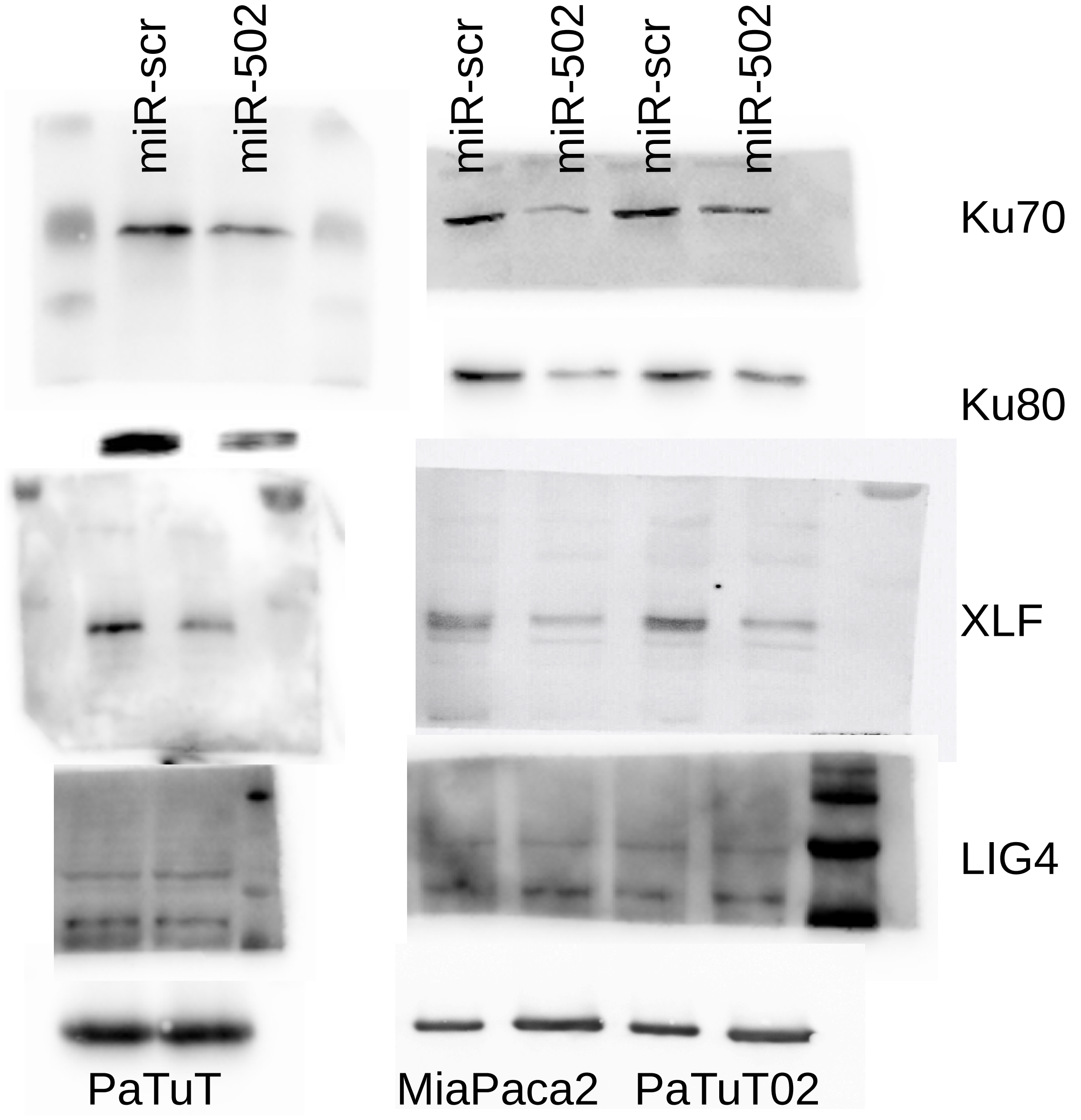

Supplementary figure 2

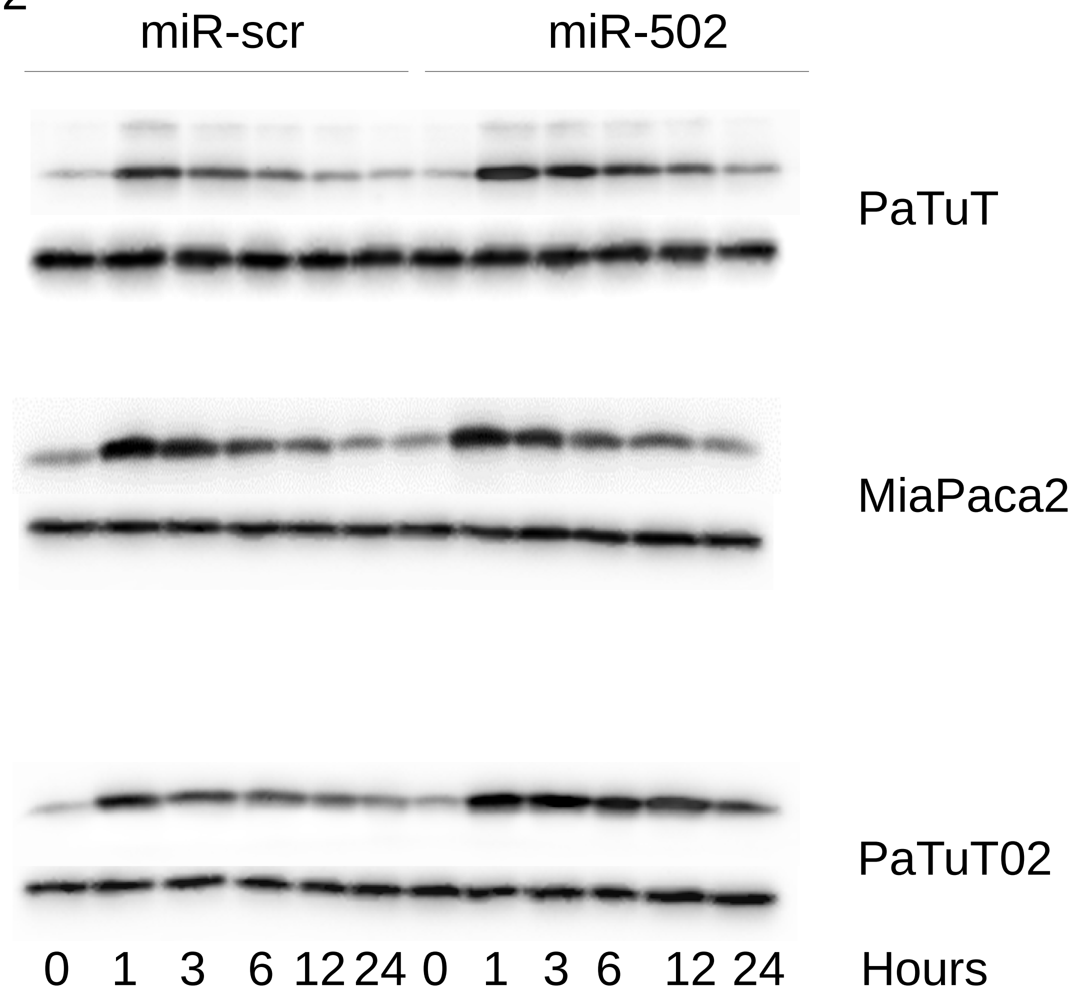

Supplementary figure 3

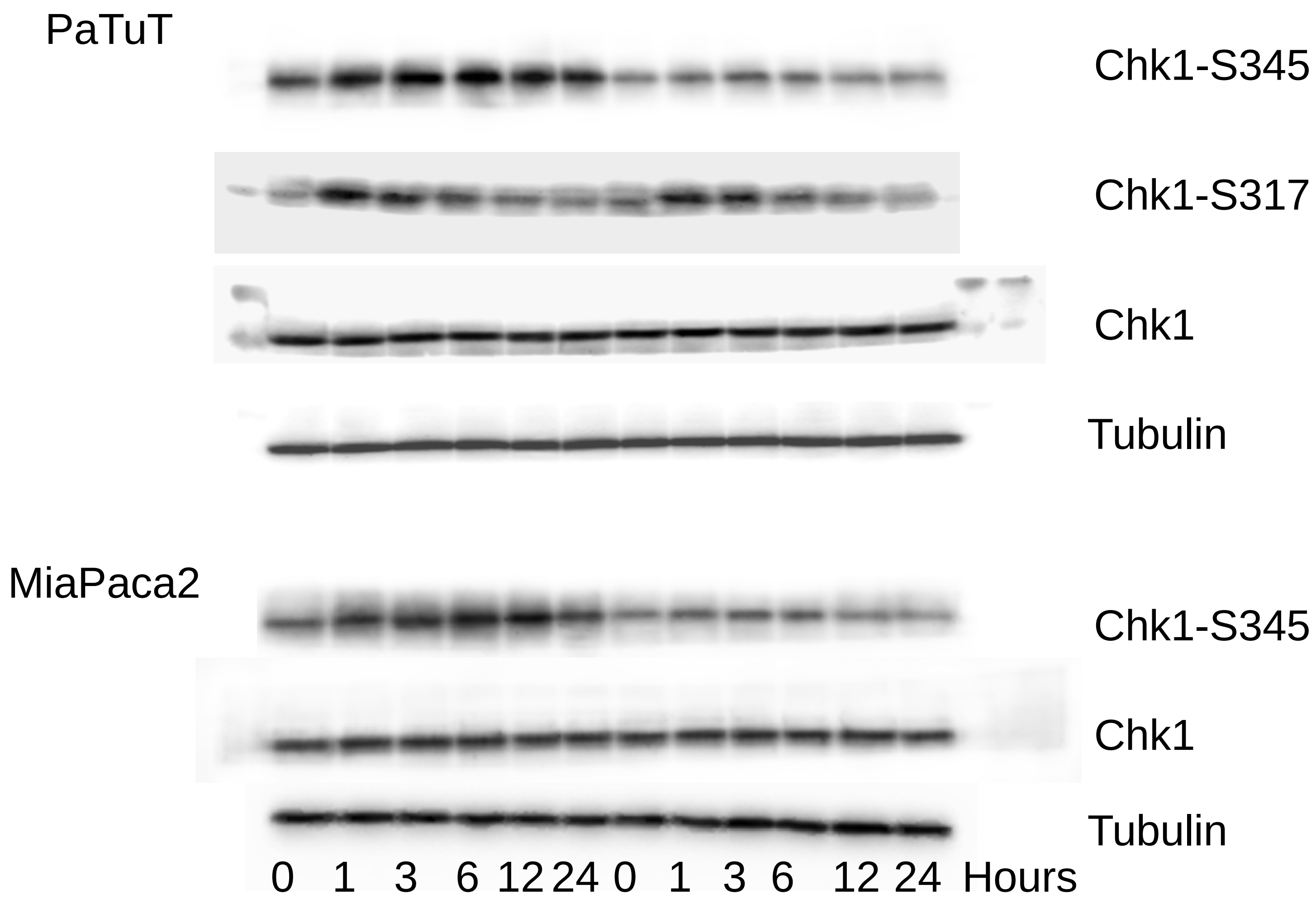

Supplementary figure 3

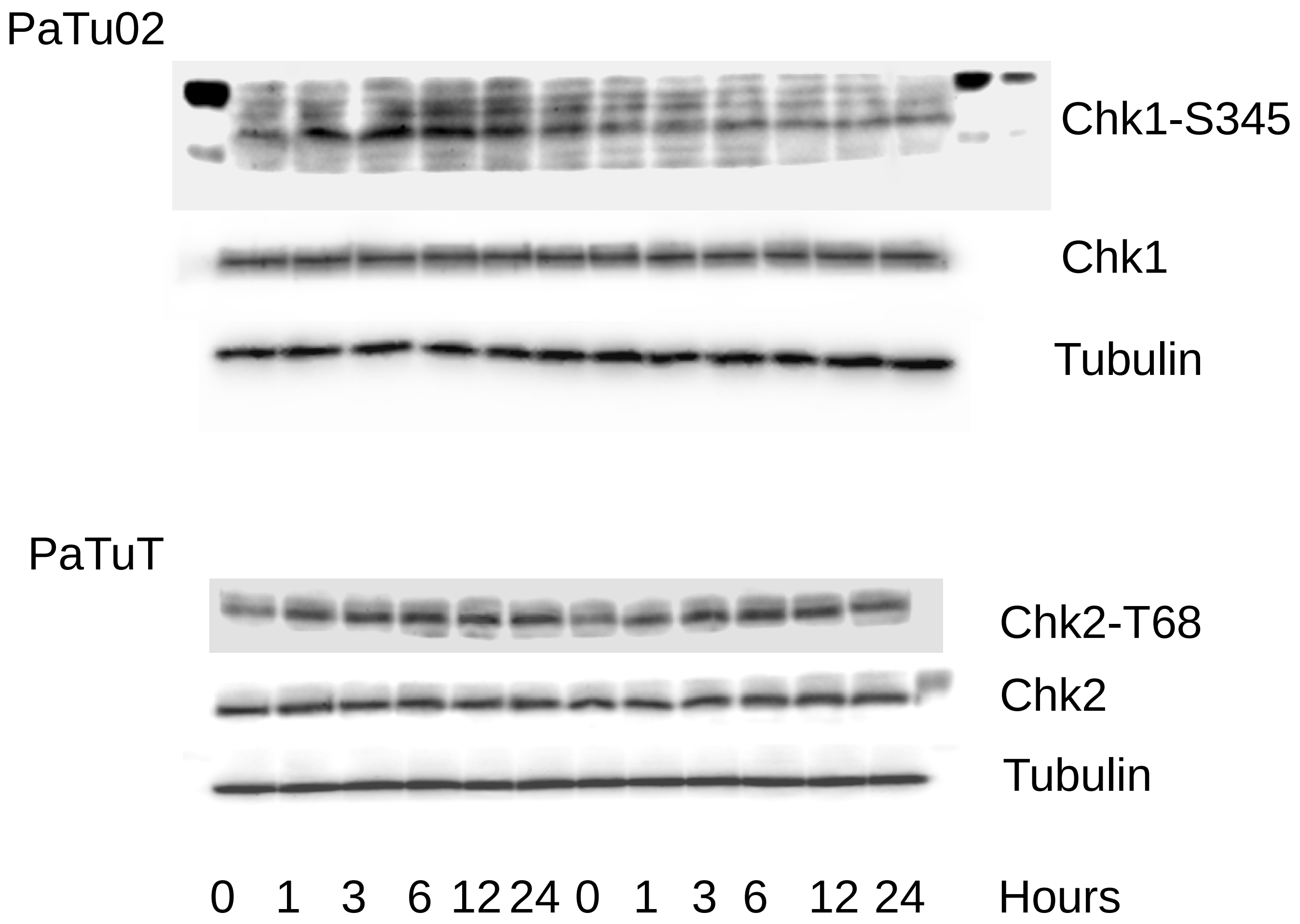

Supplementary figure 4

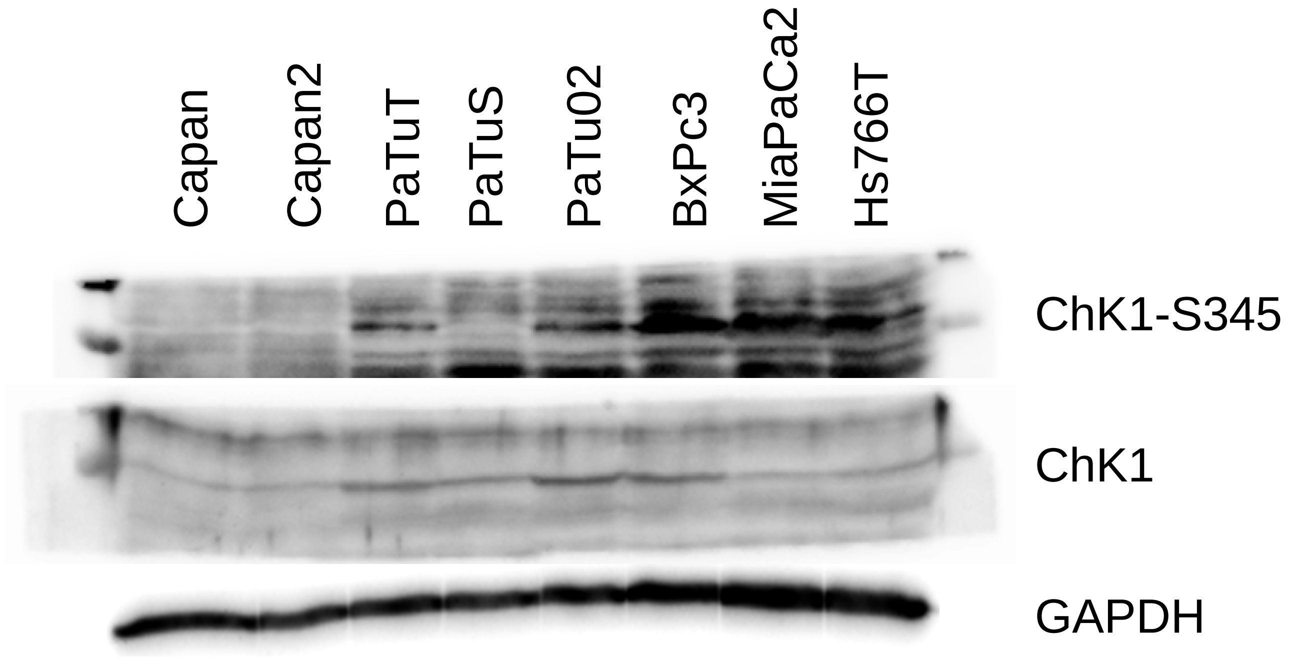

Supplement: Supplementary material-Heliyon [file mmc1.pdf]
